# Automated Analysis of the US Drought Monitor Maps With Machine Learning and Multiple Drought Indicators

**DOI:** 10.3389/fdata.2021.750536

**Published:** 2021-10-25

**Authors:** Pouyan Hatami Bahman Beiglou, Lifeng Luo, Pang-Ning Tan, Lisi Pei

**Affiliations:** ^1^ Department of Geography, Environment, and Spatial Sciences, College of Social Science, Michigan State University, East Lansing, MI, United States; ^2^ Department of Computer Science and Engineering, College of Engineering, Michigan State University, East Lansing, MI, United States

**Keywords:** USDM, machine learning, drought monitoring, logistic regression, random forest, SVM–support vector machines, drought indices

## Abstract

The US Drought Monitor (USDM) is a hallmark in real time drought monitoring and assessment as it was developed by multiple agencies to provide an accurate and timely assessment of drought conditions in the US on a weekly basis. The map is built based on multiple physical indicators as well as reported observations from local contributors before human analysts combine the information and produce the drought map using their best judgement. Since human subjectivity is included in the production of the USDM maps, it is not an entirely clear quantitative procedure for other entities to reproduce the maps. In this study, we developed a framework to automatically generate the maps through a machine learning approach by predicting the drought categories across the domain of study. A persistence model served as the baseline model for comparison in the framework. Three machine learning algorithms, logistic regression, random forests, and support vector machines, with four different groups of input data, which formed an overall of 12 different configurations, were used for the prediction of drought categories. Finally, all the configurations were evaluated against the baseline model to select the best performing option. The results showed that our proposed framework could reproduce the drought maps to a near-perfect level with the support vector machines algorithm and the group 4 data. The rest of the findings of this study can be highlighted as: 1) employing the past week drought data as a predictor in the models played an important role in achieving high prediction scores, 2) the nonlinear models, random forest, and support vector machines had a better overall performance compared to the logistic regression models, and 3) with borrowing the neighboring grid cells information, we could compensate the lack of training data in the grid cells with insufficient historical USDM data particularly for extreme and exceptional drought conditions.

## Introduction

Drought is a common, periodic and one of the costliest natural disasters that has direct and indirect economic, environmental and social impacts (Wilhite et al., 2007). These impacts become even more serious with the potential increase of drought occurrence and severity caused by climate change (Dai, 2011). A systematic and effective drought monitoring, prediction and planning system is thus crucial for drought mitigations (Boken, 2005). However, as discussed in Hao et al. (2017), drought analysis is not an easy task for a number of reasons. To start with, there is a lack of an explicit and universally accepted definition for drought since it is a multi-faceted phenomenon. Based on the variables in consideration, there are four general types of droughts, namely meteorological, agricultural, hydrological, and socioeconomic drought, for each of which different combinations of drought indices are used to characterize them (Keyantash and Dracup, 2002). Yet, there is no agreement on typical indices and their thresholds for those drought types (M. Hayes et al., 2011) since they do not work for all circumstances (Wilhite, 2000).

Although developing and choosing a proper set of physical drought indices is the basis of drought monitoring to capture the complexity and describe the consequences of drought, a composite index method has been proved to bring more success to the analysis (Hao et al., 2017). The U.S. Drought Monitor (USDM) was developed as the landmark tool in this regard as it not only uses physical drought indices, but also relies on expert’s knowledge in the information interpretation (Anderson et al., 2011). This type of composite drought monitoring, which transforms an abundant set of indicators into a sole product, is called the “hybrid monitoring approach” (M. J. Hayes et al., 2012).

The USDM was established in 1999 aiming at presenting current drought severity magnitude in the categorical means across the U.S. in a weekly map published every Thursday. In the USDM maps, drought is categorized into five categories starting from D0 (abnormally dry), to D1 (moderate drought), D2 (severe drought), D3 (extreme drought), and D4 (exceptional drought). The categories are based on a percentile approach which allows the users to interpret the drought intensity concerning the odds of event occurrence in 100 years (Svoboda, 2000). For example, D0 corresponds to a 20–30% chance for the drought to occur in ranges from 20 to 30 while for D4 it is less than 2%. For observation of the most recent map of the drought condition, refer to droughtmonitor.unl.edu, 2018.

To date, there are six main physical indicators in USDM to define the intensity of the categories: Palmer Drought Severity Index (PDSI) (Palmer, 1965), Climate Prediction Center (CPC) Soil Moisture Model Percentiles, U.S. Geological Survey (USGS) Daily Streamflow Percentiles, Percent of Normal Precipitation and Standardized Precipitation Index (SPI), and remotely sensed Satellite Vegetation Health Index (VT) along with many other supplementary indices such as the Keetch-Bryam Drought Index (KBDI) for fire, Surface Water Supply and snowpack (Svoboda et al., 2002), etc. These indices merged with other *in situ* data are jointly analyzed by experts to depict the drought categories across the country (M. J. Hayes et al., 2012).

The characteristics of the USDM which makes it a distinct effort in terms of drought monitoring are provided in Table 1. Since the uniqueness of the USDM has made it extremely popular, much attention is drawn to it from media, policy makers and managers (USDA, 2018) as a benchmark in their drought related communications and interpretations. Similarly, researchers have started using the USDM product as a reference observation to compare and validate their proposed drought monitoring and prediction methods (Gu et al., 2007; Brown et al., 2008; Quiring, 2009; Anderson et al., 2011; Anderson et al., 2013; Hao and AghaKouchak, 2014; Otkin et al., 2016; Lorenz et al., 2017a, 2017b). Although it is desirable to predict the USDM drought conditions which are in categorical format, it would not be an easy task due to the subjectivity included in the production process by the experts. A few studies (Hao, et al., 2016a; Hao, et al., 2016b) predicted the monthly average USDM drought categories using ordinal regression by integrating multiple drought indices. However, there has been no previous study using machine learning approaches to predict the USDM drought categories specifically in the original weekly format as the USDM publishes the maps.

**TABLE 1:** Uniqueness of the US drought monitoring (droughtmonitor.unl.edu, 2019; Svoboda et al., 2002).

Characteristics	Details
The first nationwide unifying drought monitoring of multiple entities	- Authors from National Drought Mitigation Center (NDMC), United States Department of Agriculture (USDA), Climate Prediction Center (CPC), and National Climatic Data Center (NCDC) have the responsibility of drawing the maps who take turns for 2 weeks
	- The authors blend the best available data from various resources for interpretation
Receives local observer’s collaboration	- More than 425 local observers such as state climatologists, National Weather Service staff, agricultural and water resources managers, and hydrologists
	- They provide drought impacts for the products using their familiarity and knowledge of the region so that the experts can depict the most accurate classification on the map
Simple and effective	- The classification system for droughts is easy to understand for public
	- Drought spatial extent, intensity, and duration are all considered
	- Flexibility with new technologies and data incorporation
Timely	- It is a weekly product which illustrates drought conditions and impacts in a timely manner

In this study, we aim to reproduce the same USDM drought analysis map over conterminous United States (CONUS) based on meteorological observations and land surface model simulated hydrological quantities through a machine learning approach and using multiple drought indicators. We apply linear and nonlinear machine learning approaches using multiple combinations of drought indices against a persistence model serving as the baseline model. The developed framework basically mimics the map synthesizing process executed by the USDM authors. This will not only test the suitability of machine learning methods in drought monitoring and prediction, but also helps us to develop tools that can translate predictions with numerical models to easy-to-understand categorical drought forecasts.

The rest of this paper is organized as follows. *Data and Methodology* elaborates the study area, data and describes the methodology. *Results and Discussion* presents the results and discussions. Finally, in the last section we summarize and conclude the findings of this study.

## Data and Methodology

**FIGURE 1:**
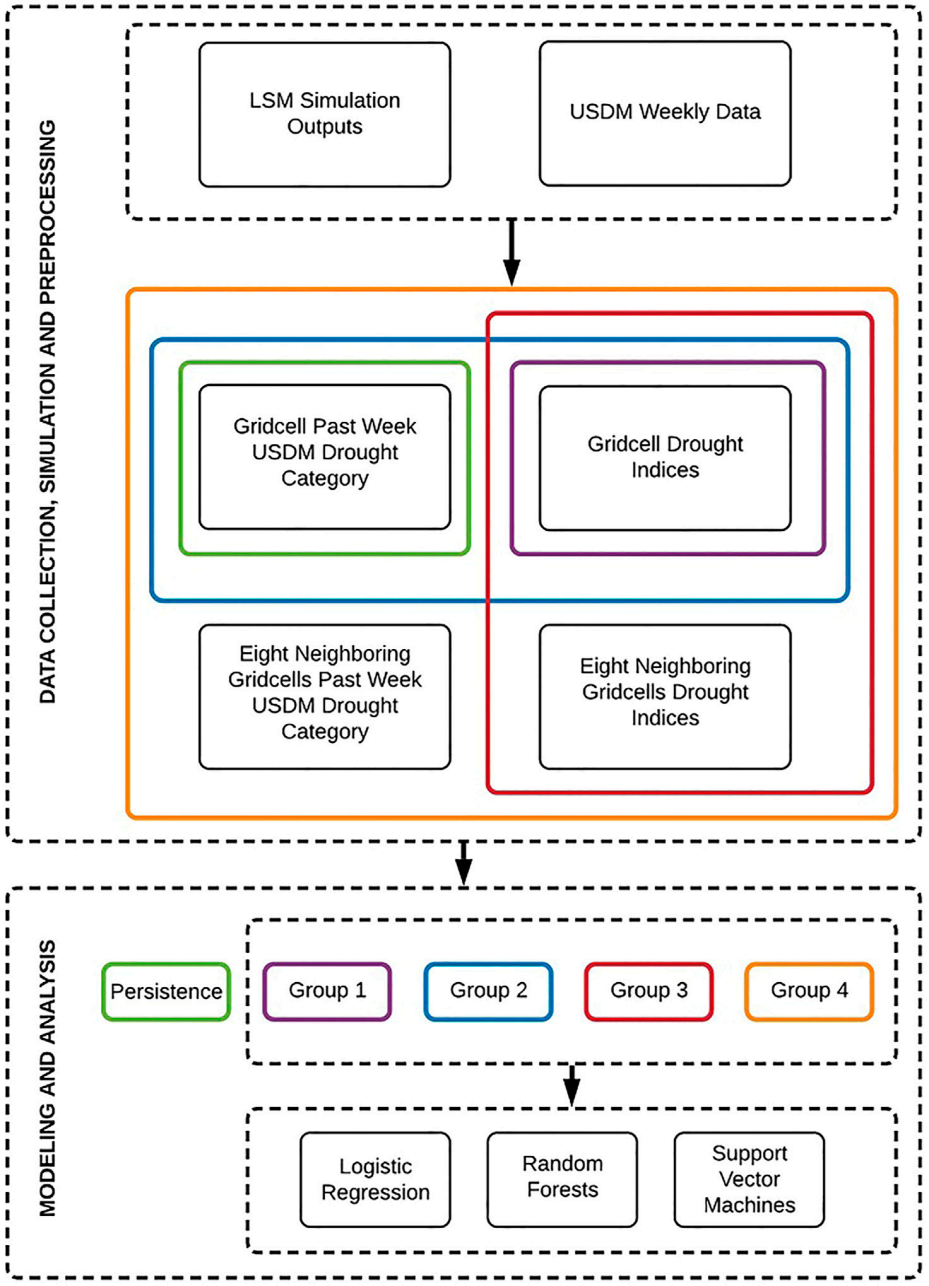
Flowchart of the proposed framework for USDM drought categories prediction.

In this section the framework of the study to reproduce the USDM drought maps is explained. The process of developing the framework is presented in Figure 1, starting from data preparation including data collection and simulation followed by data preprocessing prior to inputting into the models. Each task is explained in the following sub-sections.

As the drought indices used in our study were derived from land-surface model outputs forced by the North American Land Data Assimilation System phase 2 (NLDAS2)’s meteorological forcing fields, in this study, we deliberately designed our modeling domain to be consistent with the NLDAS2 grids. Thus, the modeling grids span the entire CONUS from 25.0625 to 52.9375° latitude and –67.0625 to –124.9375° longitude, at 1/8^º^ latitude-longitude degree resolution which forms a meshed area with 224 rows and 464 columns (Mitchell et al., 2004).

### Data Collection, Simulation, and Preprocessing

To reproduce the USDM maps, a collection of predictor variables, which correspond to drought indices were needed to predict the USDM categories. In the following paragraphs, the process of data collection, simulation, and preprocessing are described. We also explain the rationale behind the selection of each variable and how we obtained, calculated, and resampled the values for each of them prior to modeling.

#### USDM Data

The USDM weekly drought maps were retrieved from the USDM archived data at https://droughtmonitor.unl.edu/Data/GISData.aspx for the years of 2000 through 2013, starting on January 4 of 2000 and ending on December 31 of 2013, creating a total of 731 weeks of data. The USDM drought maps are vector data that outline the regions in each drought category. As the goal of this study is to reproduce the weekly USDM drought condition across CONUS, each weekly map has to be rasterized to 1/8° NLDAS2 grid. Then for every week, each grid cell is labeled as one of the five USDM drought categories or “No Drought” which makes an overall of six possible states. In the resterization process, any grid cell covering two or more different drought categories is labeled with the drought category which occupied the largest area.

#### Land Surface Model Outputs and Drought Indices

As the input variables of the actual USDM weekly report vary widely, we selected the frequently used indices in forecasting and monitoring drought. These indices are also the ones that benefit the USDM weekly map production (Anderson et al., 2011). Standardized Precipitation Index (SPI), Standardized Runoff Index (SRI), Soil Moisture Percentile (SMP), and Palmer Drought Severity Index (PDSI) are the employed indices in this study which are summarized in Table 2 and are used as the predictors of the models to predict the USDM drought categories.

**TABLE 2:** Summary of the drought indices.

Drought index	Definition	Used in This study	Source(s) of data in This study	References
Standardized Precipitation Index (SPI)	The number of standard deviations that the cumulative precipitation deficit would deviate from the long-term normalized mean. SPI value can be calculated for multiple time scales, covering the last 1, 2, 3, 4, 5, 6, 7, 8, 9, 10, 11, 12, 15, 18, 24, 30, 36, 48, 60, and 72 months	SPI for the 30, 60, and 90 days prior to the day of forecast	NLDAS-2 Forcing File A Precipitation	McKee et al. (1993)
Standardized Runoff Index (SRI)	Defined the same as SPI, except for runoff, the number of standard deviation that the percentile of cumulative hydrologic runoff would deviate over a particular duration	SRI for the 30, 60, and 90 days prior to the day of forecast	Noah3.6, NoahMP3.6, clsmf2.5 Surface, and Subsurface Outputs	Shukla and Wood (2008)
Soil Moisture Percentile (SMP)	The quantile of the current day top 1-m total soil moisture among all the data pools from the historical period over a particular onward and backward time window	Top 1-m soil moisture 29 Days Time Window	Noah3.6, NoahMP3.6, clsmf2.5 Soil Moisture Outputs	Sheffield et al. (2004)
Palmer Drought Severity Index (PDSI)	A standardized index in which the inputs of monthly temperature, precipitation and the available water capacity (AWC) of the soil are used for estimation of dryness	PDSI	Obtained from Abatzoglou (2019)	Palmer (1965)

SRI and SPI are typically calculated based on monthly data, and can be calculated for up to 72 months historical time periods. In this study, as we try to predict USDM weekly maps, we calculate the SPI and SRI based on daily data (Table 2) at 30-days, 60-days, and 90-day periods. These are the periods that prior to the day of forecast. For convenience, we still call them SPI1, SPI2, and SPI3, just to be consistent with other literature as their time scales are roughly equivalent to 1, 2, and 3 months. In order to create the indices, we first gathered the outputs of both NLDAS-2 Forcing precipitation data and the Land Information System (LIS) models (Noah-3.6, Noah-MP3.6, CLSM-F2.5) runoff and soil moisture from 1979 to 2013. The NLDAS-2 Precipitation, and LIS models hydrological runoff and soil moisture were used to calculate SPI, SRI, and SMP, respectively. It is notable that the calculations of SRI and SMP were based on the average value of three LIS models outputs. SMP values were calculated at the top 1 m for 29-days time window. More specifically, the soil moisture data of 2 weeks backward and onward time window were added to the data of the target day soil moisture to form the data pool for this date in order to compute SMP. Lastly, PDSI data was obtained from Abatzoglou (2019) in 1/24°, which then was projected and resampled to the NLDAS extents. Altogether, throughout the entire domain, every grid cell holds 731 values for each index where each was calculated for the dates that the USDM weekly maps between 2000 and 2013 were published.

#### Predictors Grouping

Different groups of predictors were used to fit the models so that the impact of different combinations of predictors on the model prediction abilities could be assessed. One of the commonly used terms in this study is the past week USDM drought category or $USDM_{t-1}$ . Here, *t* is time with weekly intervals, so *t-1* takes place a week lagging from the current time. As drought phenomena is a slow-moving process, the likelihood of switching the drought condition from current week to next week is usually low. Considering that fact, we attempt to examine the proposed models performances with inclusion or exclusion of the $USDM_{t-1}$ as an input feature, in order to find this feature importance in the prediction tasks. Moreover, by the idea of using past week drought condition as a predictor, we aim to discover how the USDM experts, aside from the use of all the physical indicators in quantifying the drought categories, would also reflect the past week drought condition as a basis in producing the current week drought map.

Toward this purpose, we defined five groups of predictors which are presented in Figure 1. It shows how different combinations of inputs (in color) supply each group of predictors. Group 1 consists the eight drought indices while Group 2 includes the past week USDM drought condition in addition to Group 1 data as one more extra predictor. In contrast to Groups 1 and 2 which solely use the target grid cell information, Groups 3 and 4 include the information of the eight neighboring grid cells as supplementary data. In other words, these Groups of data contains a three by three matrix of grid cells, centered on the target grid cell with nine times more data points. Similar to Group 1, Group 3 includes only the eight drought indices while Group 4 includes the past week USDM data of the grid cells as an additional predictor. Accordingly, one of the five groups of data is imported in the persistence model. This group of data only takes $USDM_{t-1}$ data and contributes in the baseline model. The baseline model is explained in the modeling section thoroughly.

**FIGURE 2:**
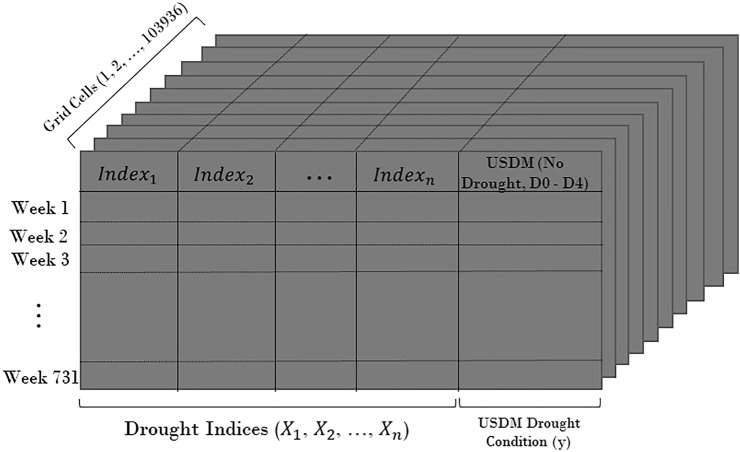
Schematic of the produced data domain.

After grouping the data, standardization of the drought indices values as well as encoding the categorical variable (i.e., $USDM_{t-1}$ ) were completed prior to inputting them in the models. Altogether, we attempt to predict the USDM drought condition labels for each grid cell by five different groups of input in the modeling. The schematic of the prepared data for the modeling in the entire domain is presented in Figure 2.

### Modeling

#### Persistence Model

In machine learning context, generally the performance of an algorithm is compared against a simple and basic method called a baseline model. The performance metric (e.g., accuracy) will then become a benchmark to compare any other machine learning algorithm against. In this study a persistence model plays the role as the baseline model. We define a persistence model as a model which assumes the current week drought condition persists in next week. In other words, the model predicts the USDM drought category of an area for a specific week as its past week drought category. In this study, the rationale for using a persistence model as the baseline model is the slow-moving nature of drought, hence the probability of a drought (or wetness) condition persisting in the next weeks could have a relatively high likelihood. Obviously, the persistence modeling for the areas with more weekly variation in drought category is subject to more prediction error. Figure 1 shows how the corresponding input data is being carried over to the persistence model.

#### Machine Learning Models

Prediction of the USDM categories is an ordinal classification problem, as it is a forced choice for the models to predict six discrete responses, No Drought, D0, D1, D2, D3, and D4. Toward this purpose, three machine learning algorithms, logistic regression, random forest classifier, and support vector machines (SVM) are selected to be examined for classification prediction.

The logistic regression model is used as a linear classification algorithm which uses the sigmoid function to limit the output of a linear equation between 0 and 1 as the probability outcome of the default class (Hosmer Jr et al., 2013). The estimation of the algorithm coefficient must be done on training data using maximum likelihood. Logistic regression is a widely used classification technique due it its computational efficiency and being easily interpretable.

Random Forest (Ho, 1995) have successfully been implemented in various classification problems (banking, image classification, stock market, medicine, and ecology) and is one the most accurate classification algorithms that works well with large datasets. The Random Forest classifier is a nonlinear classifier which consists an ensemble of decision tree classifiers. Each classifier is generated by a random set of features sampled independently from the input features, and each tree deposits a unit vote for the most suitable class to classify an input vector (Breiman, 2001). There are not many hyperparameters and they are easy to understand. Although, one of the major challenges in machine learning is overfitting, but the majority of the time this will not occur to a Random Forest classifier if there are sufficient trees in the forest and the hyperparameters, particularly the maximum depth of trees is tuned.

SVM are broadly used as a classification tool in a variety of areas. They aim to determine the position of decision boundaries that produce the most optimum class separation (Cristianini and Shawe-Taylor, 2000). In classification, a maximal margin hyper-plane separates a specified set of binary labeled training data. However, if there is no possible linear features separation, SVM employ the techniques of kernels to make them linearly separable after they are mapped to a high dimensional feature space. The two standard kernel choices are polynomial and Radial Basis Function (RBF). In this study, we use an RBF kernel in SVM classifiers since RBF kernel is more capable compared to polynomial in representing the complex relationships in data especially the synergic complexities associated with growing data.

#### 5-Fold Cross-Validation

With the use of each machine learning algorithm and group of input variables, for each grid cell in the domain we build its own specific models. In all the three modeling algorithms, choosing the optimal learning parameter(s) of the models known as “hyperparameter tuning” was performed by splitting the data to 80% training and 20% testing and executing 5-fold cross-validation on the training to select the best model. The logistic regression has only one hyperparameter, C with an L2 regularization (i.e., squared degree of coefficient as penalty term to loss function) in this study. For the Random Forest we set 200 trees as the number of estimators and then search in the parameter grid of maximum features, maximum depth, minimum samples leaf to find the optimum combination. Lastly, the hyperparameters in SVM with RBF kernel are C and γ, where C is the penalty of the objective function for misclassification and γ is the parameter of the kernel which controls the tradeoff between error of bias and variance in the model.

#### Metric of Performance Assessment

In this study, $F_{1}$ Score is selected as the metric to evaluate the model performance. $F_{1}$ Score is usually more useful than Accuracy, especially when there are uneven class distributions. This is the case in our study as in general the number of weeks that the area of a grid cell may experience the extreme (D3) or the exceptional (D4) drought is far less than the rest of the drought categories, while the number of No Drought, D0, D1, and D3 are not equal either. $F_{1}$ Score is defined as the harmonic average of precision and recall (Goutte and Gaussier, 2005):

$$Precision=\;\frac{True\;Positive}{True\;Positive+False\;Positive}$$ (1)

$$Recall=\;\frac{True\;Positive}{True\;Positive+False\;Negative}$$ (2)

$$F_{1}=\;2\;\times \frac{precision\;\times recall}{precision+recall}$$ (3)

when a larger $F_{1}$ score indicates a higher generalization ability and subsequently a higher prediction accuracy by the model. Once the Groups 1, 2, 3, and 4 input features of each grid cell are modeled and tested using the three proposed classifiers, twelve different accuracy outcomes are produced. Ultimately, all the outcomes are compared against the persistence model accuracy one by one (grid cell by grid cell) across the entire domain so that the best general combination in terms of group of features and algorithm performance is determined.

## Results and Discussion

**FIGURE 3:**
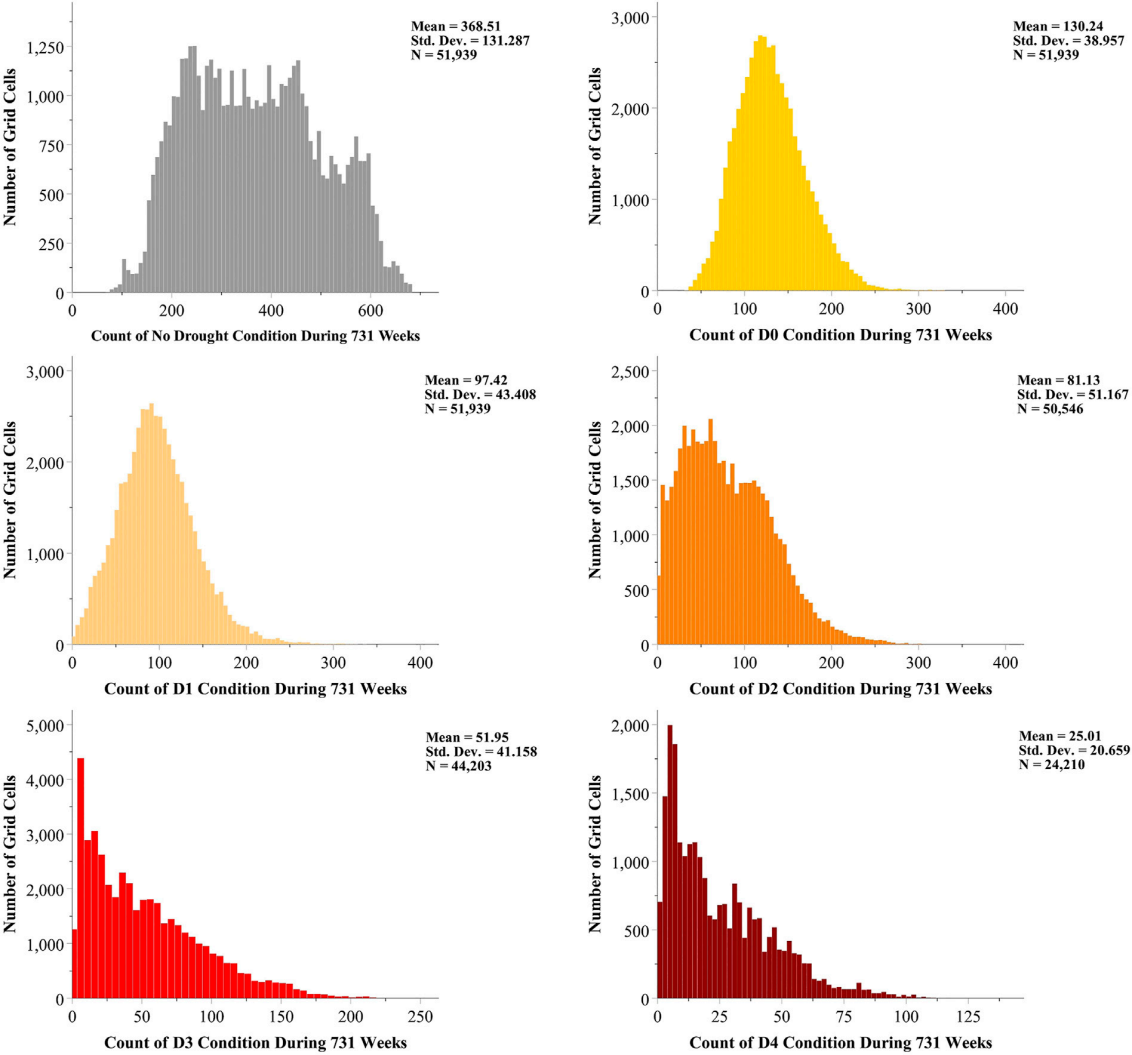
Histograms of the USDM drought categories counts across the domain in 14 years.

Our introductory analysis to the data was to explore the spread of different drought conditions across the domain during the 731 weeks. By utilizing the outcome, we can better perceive the contribution associated to the number of data points with the models prediction performance. Out of 103,936 grid cells within the domain, 51,997 grid cells never experienced any USDM drought condition during the 14 years of data which means they were always labeled as No Drought or were not in the USDM weekly maps CONUS domain. The remaining 51,939 grid cells have experienced both D0 and D1 drought categories at least once during that time period. Therefore, in our classification task, there were at least three different classes, No Drought, D0 and D1 which are to be predicted. However, for the grid cells experiencing more of the drought conditions other than D0 and D1, the prediction is a multi-class classification task of four or more classes. During 731 weeks of the USDM data, there were 50,546 grid cells experiencing D2 (as well as No Drought, D0, and D1), 44,203 grid cells experiencing D3 (in addition to No Drought, D0, D1, and D2) and 24,210 grid cells experiencing D4 (along with No Drought, D0, D1, D2, and D3) at least once. Figure 3 presents the histograms of each drought category throughout the entire domain. The included grid cells in the histograms are out of those 51,939 which have experienced more than one type of USDM drought condition. From the histograms we can observe as the drought conditions become more severe (from No Drought to D4), the grid cell mean count of the categories decrease from 369.51 for No Drought down to 25.01 for D4.

### Persistence Model

As we discussed earlier, the group of data which solely contained the $USDM_{t-1}$ was the input of the baseline predictive model known as the persistence model. The persistence model overall performance is presented in Table 3, including the minimum, maximum and mean prediction $F_{1}\;$ scores for every USDM drought category as well as the weighted average $F_{1}\;$ score.

**TABLE 3:** Persistence model descriptive statistics over the entire domain.

	Min $F_{1}$	Max $F_{1}$	Mean $F_{1}$	Std. Dev
No Drought	0.90	0.99	0.96	0.01
D0	0.42	0.96	0.81	0.08
D1	0	0.98	0.83	0.09
D2	0	0.99	0.84	0.11
D3	0	0.99	0.85	0.14
D4	0	0.99	0.83	0.20
Weighted Average	0.81	0.97	0.91	0.03

The results in Table 3 show that the persistence model prediction score for all the classes and the weighted average is relatively high. This is basically an endorsement for the slow-moving nature of drought so that a persistence model achieves such high scores at all levels.

The persistence model performs worse in the areas with more drought weekly fluctuations since an alteration in the drought condition from the current week to the next corresponds to one prediction error for the model. Furthermore, the standard deviation of the accuracies from No Drought to D4 constantly increases, yet the Weighted Average standard deviation (in Table 4) stays as small as 0.03 because of the larger weights of the less severe drought conditions in contrast to D3 and D4 categories.

**TABLE 4:** Descriptive statistics of the models performances using Group 1 input features over the entire domain.

	Logistic regression				Random forest				SVM			
	Min $F_{1}$	Max $F_{1}$	Mean	Std. Dev	Min $F_{1}$	Max $F_{1}$	Mean	Std. Dev	Min $F_{1}$	Max $F_{1}$	Mean	Std. Dev
No Drought	0.00	1.00	0.76	0.17	0.00	1.00	0.85	0.12	0.00	1.00	0.85	0.15
D0	0.00	0.78	0.20	0.18	0.00	0.92	0.55	0.13	0.00	0.94	0.60	0.12
D1	0.00	1.00	0.18	0.22	0.00	1.00	0.56	0.17	0.00	1.00	0.60	0.16
D2	0.00	1.00	0.24	0.26	0.00	1.00	0.60	0.20	0.00	1.00	0.63	0.19
D3	0.00	1.00	0.28	0.31	0.00	1.00	0.59	0.27	0.00	1.00	0.65	0.24
D4	0.00	1.00	0.44	0.39	0.00	1.00	0.63	0.34	0.00	1.00	0.70	0.29
Weighted Average	0.11	0.95	0.54	0.15	0.43	0.97	0.75	0.07	0.47	0.98	0.77	0.07

**FIGURE 4:**
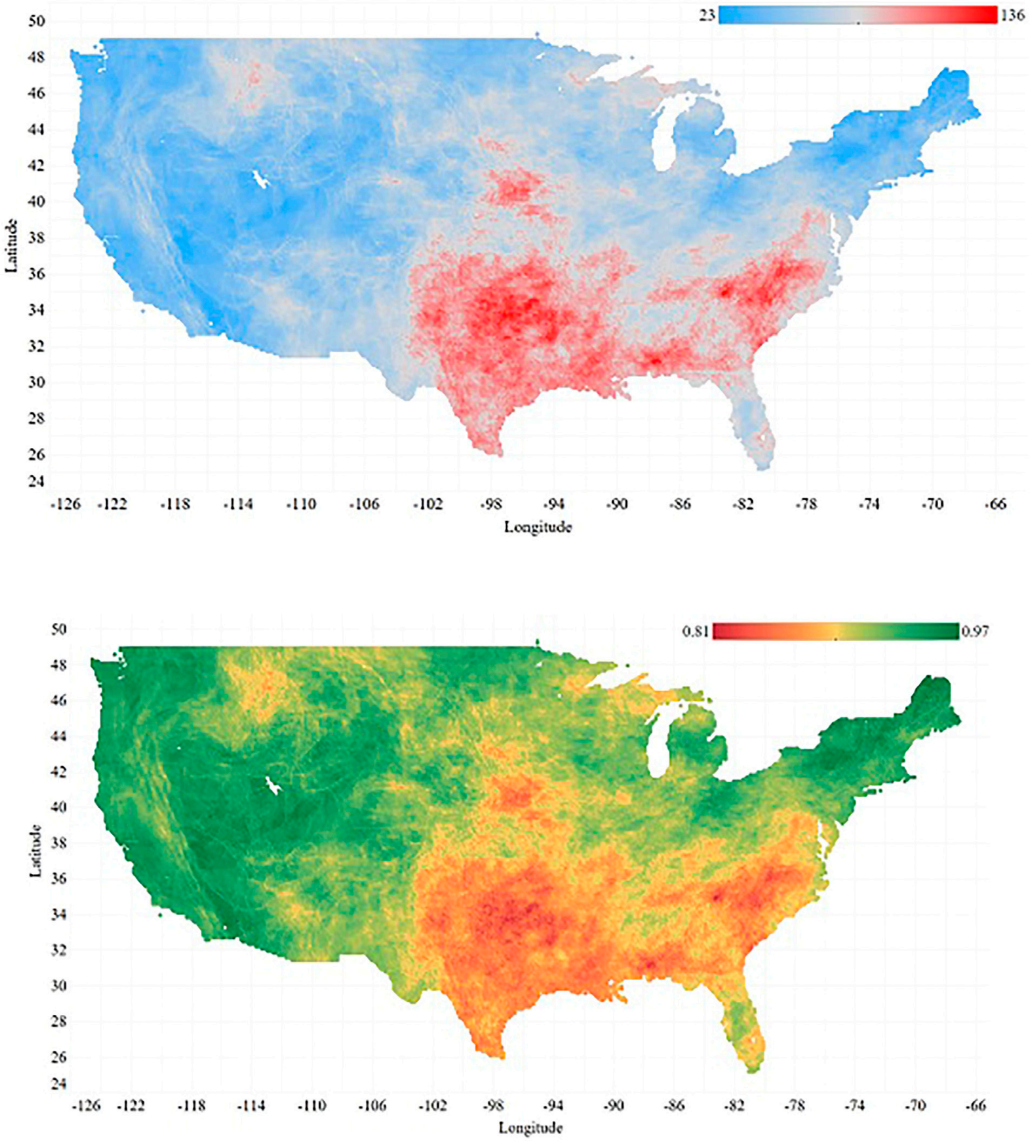
Upper Panel: Spatial presentation of the number of weekly fluctuations for each grid cell during 731 weeks (the number of changes in drought condition during 731 weeks)–Lower Panel: Spatial distribution of the persistence model weighted average F1 Score across the domain of study.

The spatial distribution of the grid cells weekly fluctuation is presented in Figure 4 (upper panel) showing the lowest variation between the USDM drought categories during 731 weeks of data is 23, while the largest is 136. As we can see, the highest weekly fluctuations are located in Southeast and Plains areas where the climate is warm temperature, humid with hot summers (Kottek et al., 2006). The lower panel in Figure 4, on the other hand, displays the persistence model weighted average $F_{1}\;$ score across the domain. As it is noticeable from the Figures, the spread patterns of colors look similar, however, in the opposite direction displaying the message that the areas with more weekly fluctuations achieve less prediction accuracy by the persistence model and vice versa.

### Machine Learning Models

#### Results for Using Group 1

In this section, we present and discuss the results of the logistic regression, Random Forest and SVM using four different Groups of input data. Table 4 contains the summary of the obtained scores for entire domain by three models by running on the Group 1 data. As we can see, the nonlinear models (i.e., Random Forest and SVM) substantially perform better than the linear model (i.e., logistic regression), while the highest scores as well as the average score are obtained by SVM for all the drought categories. However, none of the models can reach the scores that were obtained by the persistence model by any means, neither for any of the six drought classes, nor on average. Moreover, the scores standard deviations of all three models are more than the baseline model so the prediction accuracies are also less consistent.

#### Results for Using Group 2

The results of the modeling with the Group 2 data set are presented in Table 5. All three models especially the logistic regression demonstrate a great improvement over the Group 1 input feature just by adding $USDM_{t-1}$ as another variable. This indicates that the models learned to put a great weight on the extra added variable which is revealed to play an important role in terms of improving the model’s prediction capabilities. Also, the models can surpass the persistence model performance in D4 prediction score, however, on average all of them achieved an equal $F_{1}\;$ score of 0.91.

**TABLE 5:** Descriptive statistics of the models performances uing Group 2 input features over the entire domain.

	Logistic regression				Random forest				SVM			
	Min $F_{1}$	Max $F_{1}$	Mean	Std. Dev	Min $F_{1}$	Max $F_{1}$	Mean	Std. Dev	Min $F_{1}$	Max $F_{1}$	Mean	Std. Dev
No Drought	0.00	1.00	0.94	0.13	0.00	1.00	0.94	0.13	0.00	1.00	0.94	0.14
D0	0.00	1.00	0.81	0.10	0.00	1.00	0.80	0.11	0.00	1.00	0.81	0.10
D1	0.00	1.00	0.82	0.14	0.00	1.00	0.81	0.15	0.00	1.00	0.82	0.15
D2	0.00	1.00	0.83	0.18	0.00	1.00	0.83	0.17	0.00	1.00	0.83	0.17
D3	0.00	1.00	0.83	0.23	0.00	1.00	0.83	0.22	0.00	1.00	0.84	0.22
D4	0.00	1.00	0.84	0.26	0.00	1.00	0.85	0.25	0.00	1.00	0.85	0.26
Weighted Average	0.77	0.99	0.91	0.03	0.75	0.99	0.91	0.03	0.75	0.99	0.91	0.03

During the model training with Group 1 and 2 data, the range of the $F_{1}$ scores of the Random Forest and SVM varied from 0.99 to 1 which was almost perfect. However, on the testing data, the scores dropped down to 0.91 to show an overfitting problem. This type of challenge sometimes happens in nonlinear models when the number of data points compared to the number of features are small even though a cross validation is used. By observing the histograms in Figure 3, we could find the reason in the average count of D3 and D4 drought categories which are usually low. With using 80% of data in training even though randomly selected, the chances of a model seeing fewer of those categories during learning process become higher. This causes the models memorize instead of learn so while testing, the scores are not as promising as training.

With the use of Group 2 data in the modeling, on average in 31,732 grid cells (61% of the domain) logistic regression performed better than or equal to the persistence model. This is the case for the Random Forest model in 27,139 grid cells (52% of the domain) and in 31,085 grid cells (60% of the domain) for the SVMs. Adding the past week information to the data, helped the models to improve their prediction accuracy, however, it was still challenging to be assertive about outperforming the baseline model. With the presumption that lack of data point may be the cause of underperformance, we tried the Groups 3 and 4 in the models so that we could possibly find out whether there would be any improvement in prediction accuracy.

#### Results for Using Group 3

The performance of the machine learning models without $USDM_{t-1}$ label as the predictor, yet with borrowing the neighboring grid cells which created Group 3 data were examined and are summarized in Table 6.

**TABLE 6:** Descriptive statistics of the models performances using Group 3 input features over the entire domain.

	Logistic regression				Random forest				SVM			
	Min $F_{1}$	Max $F_{1}$	Mean	Std. Dev	Min $F_{1}$	Max $F_{1}$	Mean	Std. Dev	Min $F_{1}$	Max $F_{1}$	Mean	Std. Dev
No Drought	0.00	0.98	0.78	0.14	0.00	1.00	0.93	0.06	0.00	1.00	0.94	0.08
D0	0.00	0.72	0.24	0.16	0.07	0.96	0.79	0.07	0.00	0.99	0.83	0.06
D1	0.00	1.00	0.21	0.20	0.00	1.00	0.79	0.08	0.00	1.00	0.83	0.07
D2	0.00	1.00	0.28	0.24	0.00	1.00	0.80	0.11	0.00	1.00	0.84	0.09
D3	0.00	1.00	0.31	0.30	0.00	1.00	0.80	0.15	0.00	1.00	0.84	0.13
D4	0.00	1.00	0.44	0.36	0.00	1.00	0.78	0.24	0.00	1.00	0.83	0.20
Weighted Average	0.17	0.93	0.55	0.14	0.61	0.99	0.87	0.05	0.63	1.00	0.90	0.05

The weighted average accuracy of the logistic regression dropped significantly once again when the past week information predictor was eliminated. Despite the importance of the eliminated predictor, the nonlinear models, Random Forest and SVM could sustain fairly close to the persistence model on average but still lower, with 0.87 and 0.90 $F_{1}\;$ prediction score, respectively. The results showed that the SVM model with Group 3 data could predict better than the persistence model for D0, while it had an equal score but less standard deviation for D1 and D2, and an equal score and standard deviation for D4.

When compared the weighted average $F_{1}\;$ score across the entire domain, the logistic regression could not surpass the persistence model prediction scores in any of the grid cells, while the Random Forest and SVM were successful in 17,385 (33% of the grid cells) and 27,743 (53% of the grid cells), respectively. The results of modeling with Group 3 indicates that by employing the neighboring grid cells data and consequently a larger training set, we could improve the models, particularly the nonlinear ones, to capture the relationships between the variables and drought categories more precisely. However, the feature $USDM_{t-1}$ still illustrates a stronger impact than the size of training data when Group 2 and 3 are compared side by side.

#### Results for Using Group 4

The results of using Group 4 dataset in the modeling are presented in Table 7. Compared to the Groups 1, 2, and 3 results, there is a noticeable improvement in $F_{1}\;$ score, for the Random Forests and SVM. The logistic regression performs slightly better than the persistence model in the prediction score for the categories, however the $F_{1}\;$ weighted average scores are equal. On the other hand, the Random Forest and SVM outperform the persistence model in all the categories and $F_{1}\;$ weighted average scores with the highest scores equal to 0.96 achieved by the SVM. Using Group 4 of data, indicates that borrowing the neighboring grid cells information and including $USDM_{t-1}$ , could certainly and significantly help the models learning curve to improve. Clearly, the lack of data points was preventing the models to capture a more comprehensive pattern while just using one single grid cell data.

**TABLE 7:** Descriptive statistics of the models performances using Group 4 input features over the entire domain.

	Logistic regression				Random forest				SVM			
	Min $F_{1}$	Max $F_{1}$	Mean	Std. Dev	Min $F_{1}$	Max $F_{1}$	Mean	Std. Dev	Min $F_{1}$	Max $F_{1}$	Mean	Std. Dev
No Drought	0.00	1.00	0.96	0.05	0.00	1.00	0.98	0.04	0.00	1.00	0.98	0.06
D0	0.36	0.96	0.81	0.08	0.51	0.99	0.90	0.04	0.61	1.00	0.93	0.03
D1	0.00	1.00	0.83	0.09	0.00	1.00	0.91	0.05	0.00	1.00	0.93	0.04
D2	0.00	1.00	0.85	0.11	0.00	1.00	0.92	0.08	0.00	1.00	0.94	0.07
D3	0.00	1.00	0.87	0.14	0.00	1.00	0.92	0.11	0.00	1.00	0.93	0.10
D4	0.00	1.00	0.85	0.20	0.00	1.00	0.90	0.18	0.00	1.00	0.91	0.17
Weighted Average	0.82	0.98	0.91	0.03	0.86	1.00	0.95	0.02	0.87	1.00	0.96	0.01

By looking into the one by one obtained $F_{1}\;$ weighted average scores for each grid cell across the domain, on average in 10,794 grid cells (21% of the domain) the logistic regression performs worse than the baseline model, whereas in only 419 grid cells (0.8% of the domain) the random forest performs worse than the persistence. The SVM with the best results, misclassified just 18 grid cells with 1 percent difference weighted average score compared to the persistence model. Aside from those 18 points with -0.01 accuracy difference, the rest of them vary between 0 and 0.13. Recall from Figure 4, it was observed that in the Southeast and Plains areas the persistence model performs worse (i.e., higher weekly fluctuation), and by looking into the results of the SVM model, it was where a larger difference in the prediction accuracy between the baseline and out machine learning model was noticed. We will discuss more about the comparison of the models predictions against the persistence model in boxplots later on in this section, although the purpose of outperforming the persistence model by the machine learning models has been met.

#### Side-By-Side Boxplot Comparison of the Model Performance Using Different Groups of Data

**FIGURE 5:**
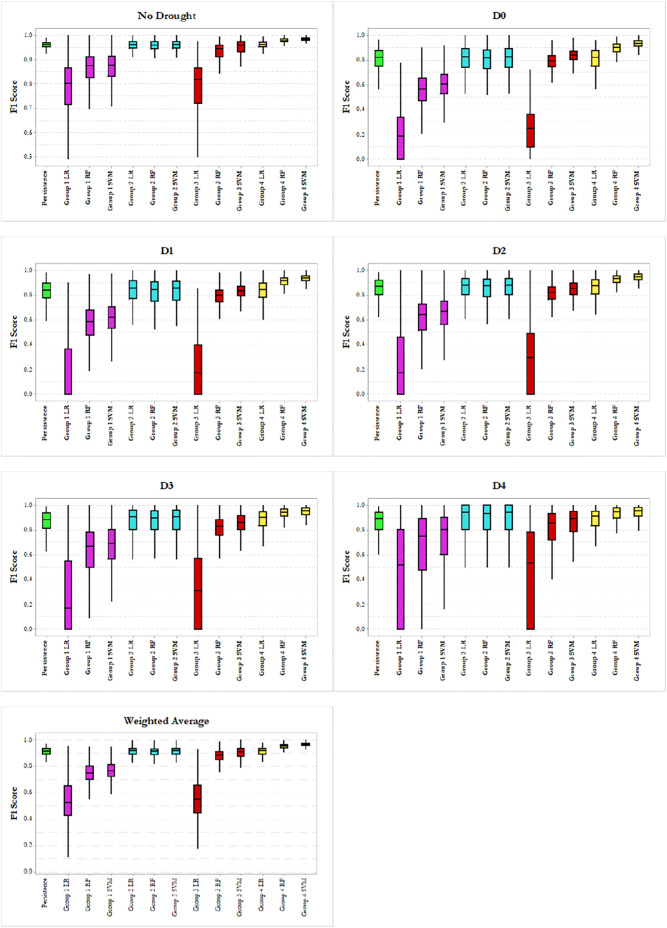
Side by side comparison of the model’s overal and for each drought category performances.

In this section, we present and discuss the performances of all the 13 different types of modeling in this study, next to each other in the format of boxplots. Figure 5 provides a side-by-side overall performance of the models (i.e., weighted average) as well as the results of the models for each USDM category. In the boxplots, the box middle line, bottom line and top line are the median, 25th percentile and 75th percentile, respectively. The whiskers extend 1.5 times the height of the box (Interquartile range or IQR), and the points are extreme outliers which are three times greater than the IQR. From the Figure Weighted Average panel, we could clearly find out that the USDM drought labels were better predicted by the nonlinear functions in terms of accuracy and deviation. The linear model fulfilled a meaningfully better prediction with the presence of the $USDM_{t-1}$ information as a predictor (Groups 2 and 4). The importance of this predictor can be observed by comparing the Groups 2 and 3 results, while modeling with Group 2 could obtain better results than Group 3, even with using fewer number of data points. The detected pattern in the overall performance can be observed in all the six drought categories, where Group 4 performs the best, followed by Group 2, then Groups 3 and lastly Group 1.

In terms of feature importance in the models, both the logistic regression and random forest commonly recognized PDSI as the most important predictor in Group 1 and Group 3, while in Groups 2 and 4, $USDM_{t-1}$ received the largest coefficient, followed by PDSI as the second most important feature. The importance of the rest of the features in the models were relatively close to each other. Unfortunately, as the RBF kernel in SVM transforms the features into a high dimensional space, the implicit transformation does not allow us to obtain the feature importance.

#### Visual Comparison of the Models Over CONUS

**FIGURE 6:**
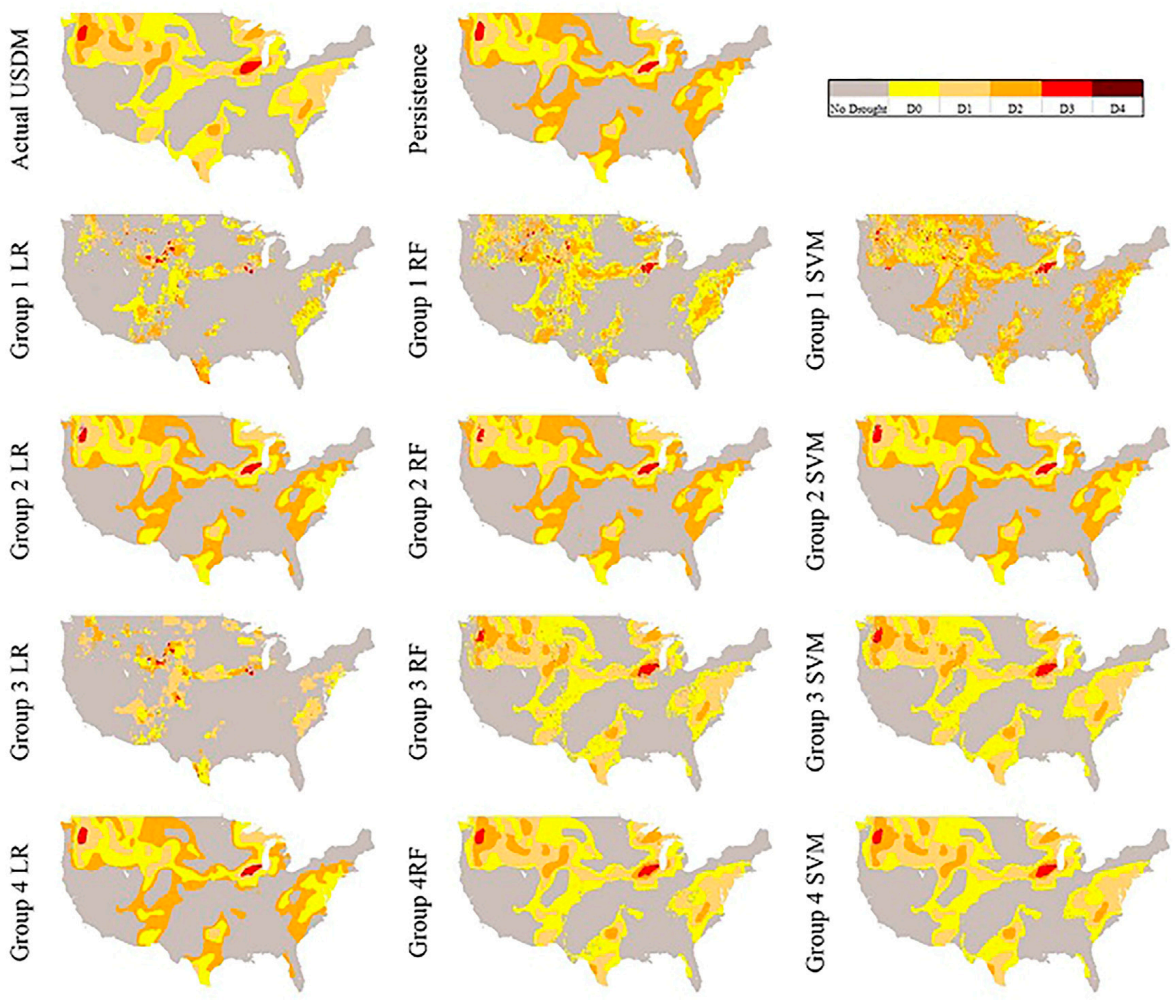
Produced maps April 10, 2005 by each model.

**FIGURE 7:**
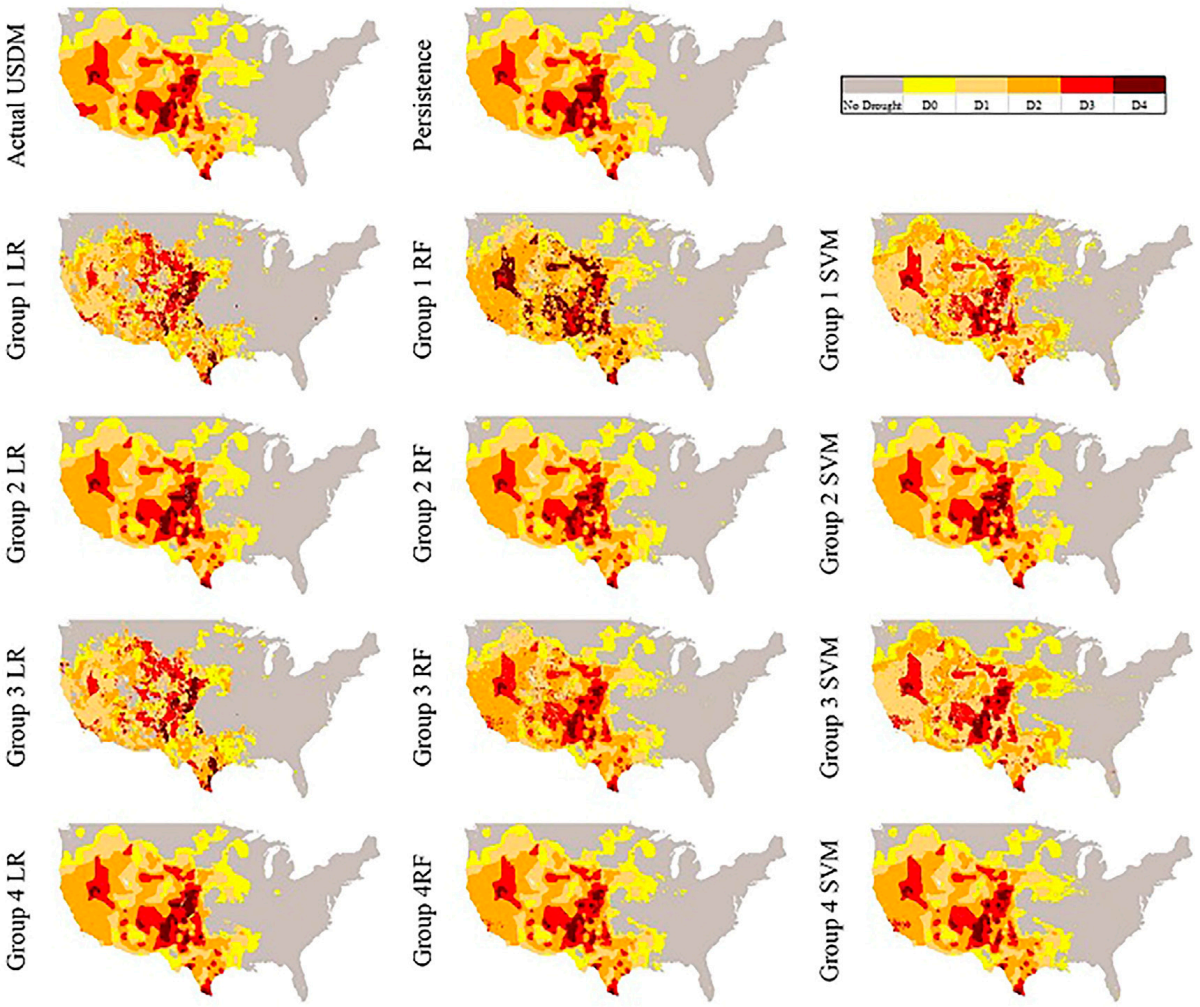
Produced maps of 08/13/2013 by each model.

For a better illustration in comparing the reproduced maps by the models and the actual USDM map, we also selected two random dates. In Figure 6, the actual USDM map is not experiencing any D4 but two spots of D3 in Midwest and Northwest regions. Figure 7, however, shows larger and more scattered areas of D3 and D4 across the domain.

In Figure 6, the persistence model can closely catch D3 areas however, it does not perform well in predicting the D0, D1, and D2 while the large areas of D0 are replaced with D1 and D2. This is possibly due to precipitations during the past week generated map date (9/27/2005) and the date of this map (April 10, 2005) in which has made those areas drought severity one category less extreme. The generated maps from Groups 1 and 3 models do not look well reproduced except Group 3 RF and SVM, however, both still are not as smooth as expected. The entire Group 2 map plus Group 4 LR are very similar to the persistence model map which means the models are heavily relying on the $USDM_{t-1}$ as their predictors. Finally, the best performing model, Group 4 SVM is able to generate very similar map to the actual USDM map followed by Group 4 RF as the second-best model. If pay a closer attention, there is a slight difference between Group 4 RF and SVM in which RF is still mispredicting few spots and does not look as smooth on the map.

Figure 7 has a relatively similar persistence model to the actual USDM map except a few small areas such as not being able to recognize an D3 area in California and replacing a No Drought region in Indiana with D0. As it can be seen, the models in Groups 1 are not doing well, however, there is a significant improvement once the models are fed with $USDM_{t-1}$ as another feature in Group 2. The maps of Group 3 models are not as smooth, but we can see the above-mentioned areas that the persistence model was not able to catch are relatively being recognized by them especially by the SVM model. Lastly, the best performing model is Group 4 SVM which was able to produce almost as similar as the actual USDM map.

**FIGURE 8:**
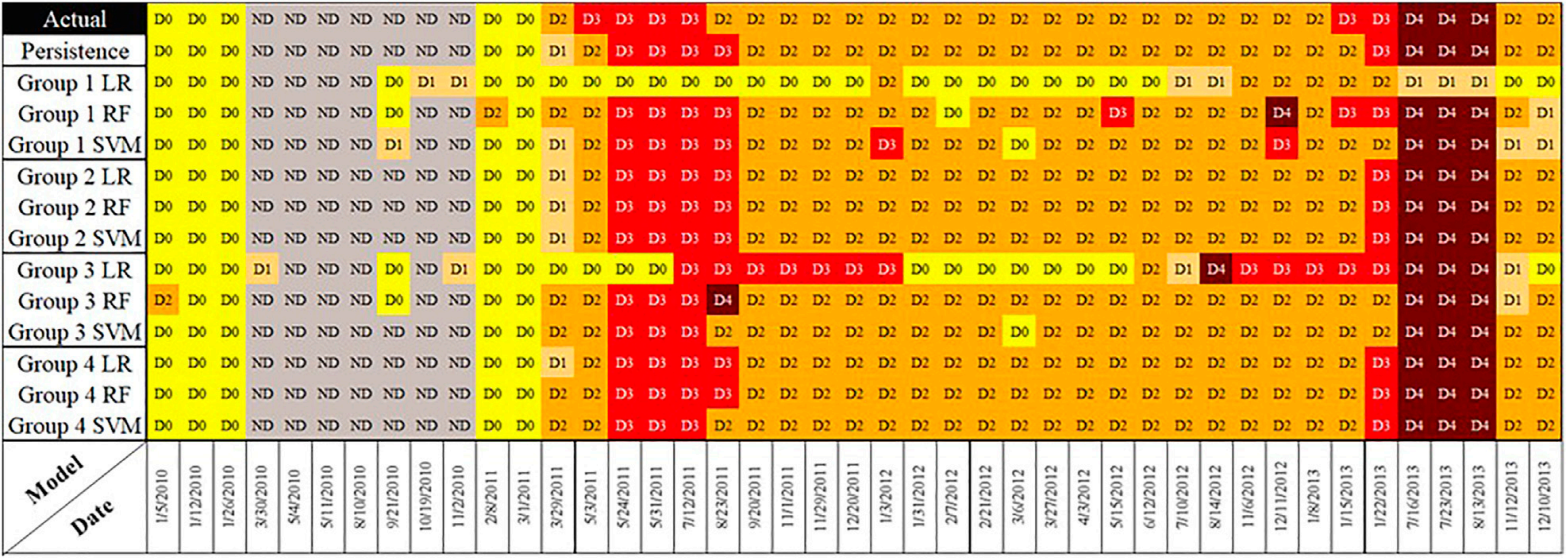
Time series of test data of grid cell located in (35.0629, −105.3130) New Mexico.

As the last step, in order to show a more in detail models comparison instead of the overall average performance, we selected a random sample grid cell located at the latitude of 35.0630, and longitude–105.3130 (appeared to be in New Mexico) and put the test data from the years 2010–2013 in a time series graph. Figure 8 presents the actual test data into the models and each model prediction. It is notable that the graph is an ordered time series of the test data points, but the dates are not consecutive due to random selection of training and test set, while the weeks in between were used as training data for the models. Similar to the above generated maps, here the Group 2 models are significantly relying on the $USDM_{t-1}$ feature as whenever the persistence does or does not predict correctly, Group 2 models are predicting accordingly. Group 1 models and Group 3 LR are the least consistent models, while Group 3 RF and SVM showed a fairly good performance even though they were not using $USDM_{t-1}$ . Group 4 models show the best modeling results especially Group 4 SVM by being able to predict the date 8/23/2011 correctly where the majority of the models failed.

## Conclusion

Our proposed framework successfully reproduced the USDM drought categories using multiple drought indices and machine learning algorithms that are logistic regression, Random Forest and SVM. The framework was compared to a persistence model as the baseline model in which it was assumed that current week drought condition would persist in next week. As this study was a classification task, the machine learning models were evaluated by their overall prediction scores as well as each class prediction score. Although, in terms of prediction accuracy, there was not much room left for improvement by the baseline model, our proposed framework could outperform it by testing different scenarios of the data inputs and machine learning algorithms to find the best combination.

We found out that employing the past week drought data as a predictor in the models played an important role in achieving high prediction scores especially for the logistic regression. The nonlinear models, Random Forest, and SVM suffered less without the use of that predictor in terms of prediction score. Furthermore, taking the neighboring grid cells information into account, could compensate the lack of data points for training the models. It was essentially a spatially compensation of the USDM data due to temporal shortage (731 weeks). Training the models faced the lack of data problem particularly for the categories D3 and D4. In some grid cells when the number of D3 and D4 were smaller than the number of the folds in cross validation (i.e., 5 in this study) as well as random selection of training and test splits, technically some folds could not contain those categories during the learning process which resulted in poor predictive skill.

Future works could be the examination of a multi-task learning approach which works well with limited data by leveraging information from nearby locations. Also, since we have been successful in being close to mimicking the USDM experts drought categories synthesizing, this methodology could be used in an automated system in generating the weekly maps. The system would be using LSMs to produces the outputs which are needed to calculate the drought indices which represent meteorological, agricultural, and hydrologic drought. Thereafter by creating the indices for the target day that the map is going to be published and using the past week drought condition as another variable, the SVM model as the best performing model in this study would predict the drought conditions across the entire United States.

## Data Availability

The raw data supporting the conclusions of this article will be made available by the authors, without undue reservation.
